# Reconstruction of extensive jaw defects induced by keratocystic odontogenic tumor via patient-customized devices

**DOI:** 10.1186/s40902-015-0038-9

**Published:** 2015-10-19

**Authors:** Seok-Yong Park, Young-Jo Shin, Chul-Hoon Kim, Bok-Joo Kim

**Affiliations:** Department of Oral and Maxillofacial surgery, Dong-a University Medical Center, Dongdaesin-dong 3-ga, Seo-gu, Busan, 602-715 South Korea

## Abstract

Keratocystic odontogenic tumors can occur in any area of the maxilla or mandible. According to their size, location, and relations with surrounding structures, they are treated by cyst enucleation or enucleation after either marsupialization or decompression. Enucleation is performed when cysts are not large and when only minor damage to adjacent anatomical structures is expected. Although marsupialization and decompression follow the same basic bone-regeneration principle, which is to say, by reducing the pressure within the cyst, the former leaves a large defect after healing due to the large fistula necessary to induce the conversion of the cyst-lining epithelia to oral epithelia; the latter leaves only a relatively small defect, because of the continuous washing carried out by means of a tube inserted into a small hole in the cyst. In the latter case too, a decompressor appropriate for the focal position is required, owing to the importance of maintaining the device and controlling for oral hygiene. We report herein decompression treatment with a patient-customized device for an extensive cyst in the anterior region of the mandible.

## Background

Keratocystic odontogenic tumors can occur in any area of the maxilla or mandible. They have a remarkable growth capacity and manifest extensive bone resorption, showing a superior overall growth performance to other odontogenic cysts.

According to cysts’ size, location, and relations with surrounding structures, they are treated by enucleation, conservative treatment, or enucleation after conservative treatment. Marsupialization and decompression are included in the conservative treatment. Enucleation is performed when cysts are not large and when only minimal damage to adjacent structures is expected. Marsupialization and decompression are performed when primary enucleation is not easy, as in cases of large intraosseous cysts, or when there is significant cause for concern about damage to adjacent structures [1, 2].

Although marsupialization and decompression are used interchangeably in many articles, they have different technical meanings. Decompression implies any means taken to reduce the pressure from within a cyst. Marsupialization in its true sense entails the conversion of the cyst into a pouch, and this implies the creation of a sizable stoma or opening that has the ability to maintain itself. Marsupialization, in short, is a means of cyst decompression [3].

In the following pages, we report the decompression of an extensive mandibular keratocystic odontogenic tumor using mini plates and 16G spinal needles.

## Case presentation

**Fig. 1:**
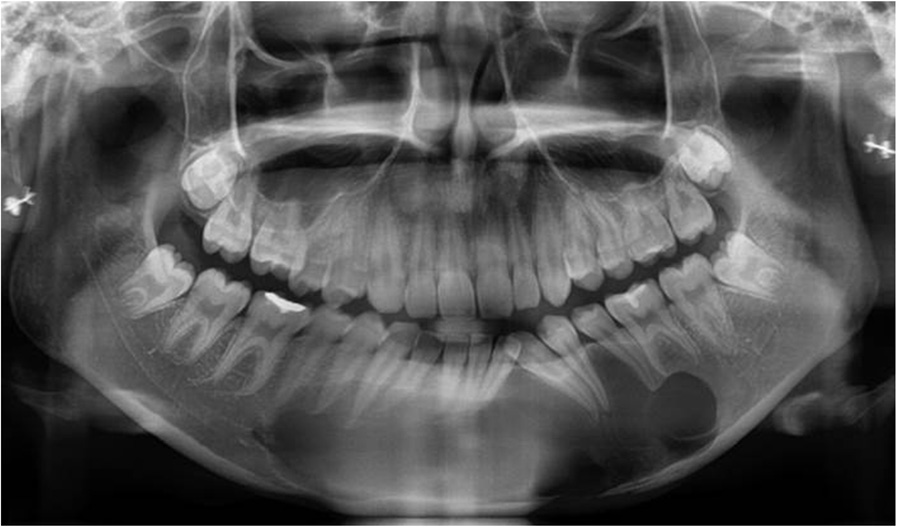
Radiographic findings at the initial visit. A well-defined radiolucent lesion is observed in the mandible. The roots of the tooth in the lesion were not resorbed, but the displacement of tooth was evident

**Fig. 2:**
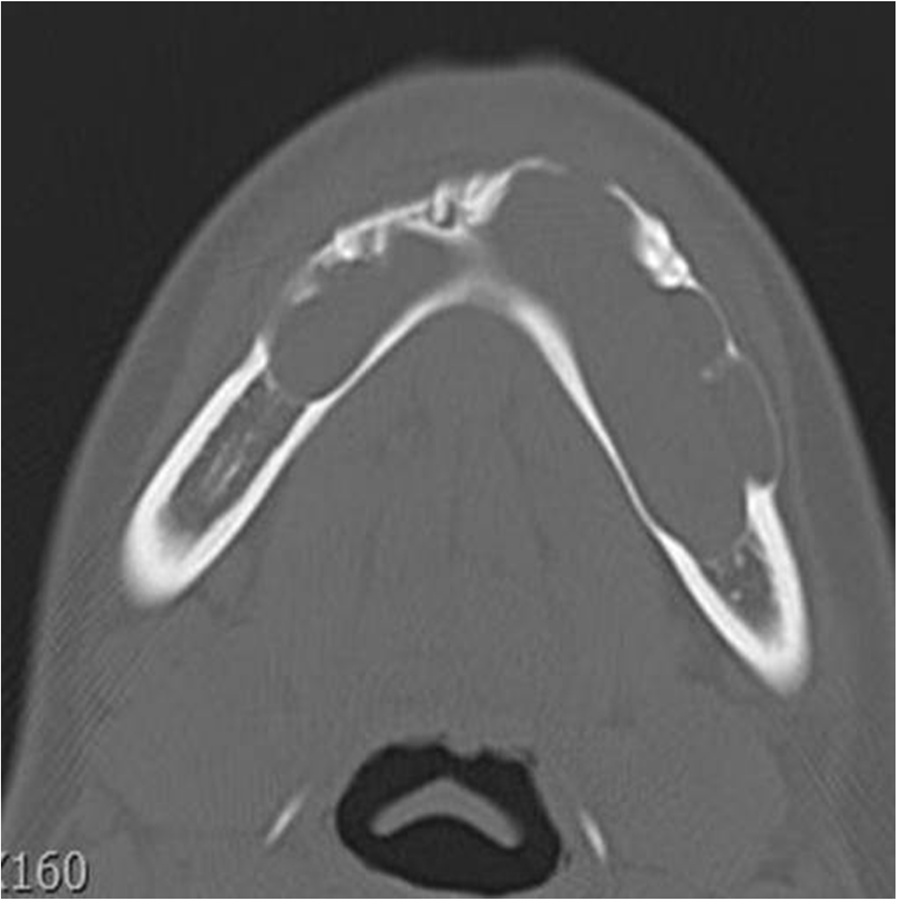
Computerized tomogram image of the mandible at the initial visit. Expansible cystic mass was found in the mandible, and the buccal cortex of the mandible showed erosive change

A 19-year-old female visited our clinic complaining of malocclusion of mandibular dentition. There was no mandibular swelling or pain. However, panoramic radiography revealed a huge well-defined radiolucent lesion, and teeth displacement from #36 to #45 was observed. The labial cortical bony walls were moderately thinned and deviated, penetrating to the labial side of the mandible (Figs. 1 and 2).

**Fig. 3:**
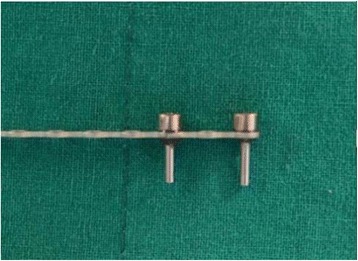
Two 16G spinal needles adhered to the Ø2.0 mini plate

The patient had no systemic problems, and because the cystic lesion had invaded to the inferior alveolar nerves, we chose enucleation after continuous drainage over cyst enucleation. A decompression device durable enough for long-term placement was required, because long-term drainage was expected, and there was concern about the possibility of continuous falling-outs, replacements, and infection if using a rubber tube. As a decompression device, 16G spinal needles were attached to a Ø2.0 mini plate with light-cured resin after forming two uneven holes using a round bur (Fig. 3).

It was possible to reduce the operation time by pre-surgical adjustment of the mini plate according to the patient’s dimensions after determining the position of the decompressor. The operation was performed under general anesthesia. The customized decompressor was attached below the mandibular anterior teeth. The attachment site had already been determined by confirmation of healthy bone on mandibular computerized tomogram (CT) images. For fixation, two mini plates were attached at the inferior border of the anterior mandible.

**Fig. 4:**
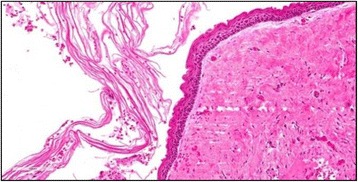
Histologic findings on cyst (hematoxylin and eosin stain, original magnification ×200). Focal epithelial interruption associated with chronic active inflammation

A tissue biopsy performed at the time of surgery indicated a keratocystic odontogenic tumor (Fig. 4). Self-cleaning of the holes in the mini plate was indicated by the 21G needle every day at the time of hospital discharge. Follow-up was performed at 1, 2, 3, and 6 months.

**Fig. 5:**
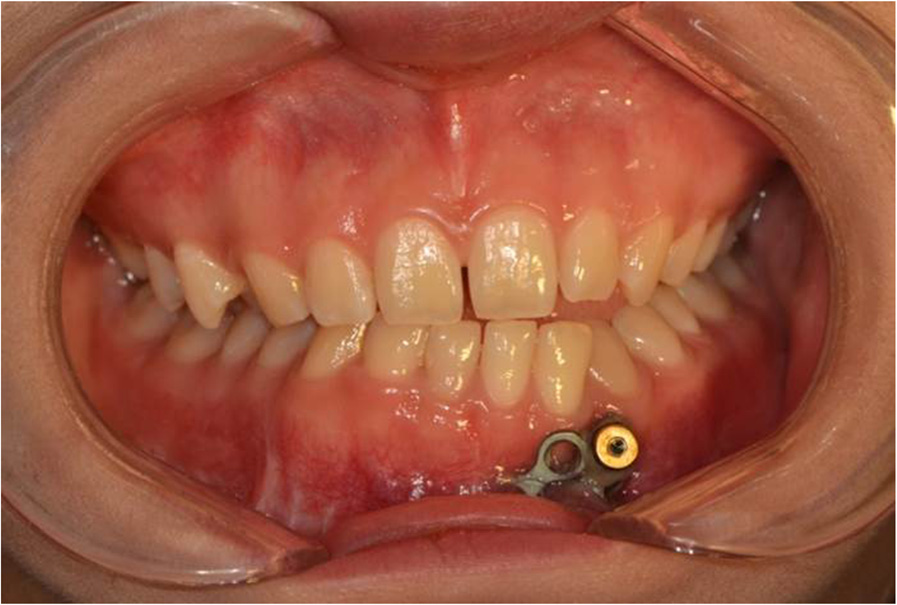
Intraoral view at 1 month postoperatively. A drain had been lost, but the other was well maintained

**Fig. 6:**
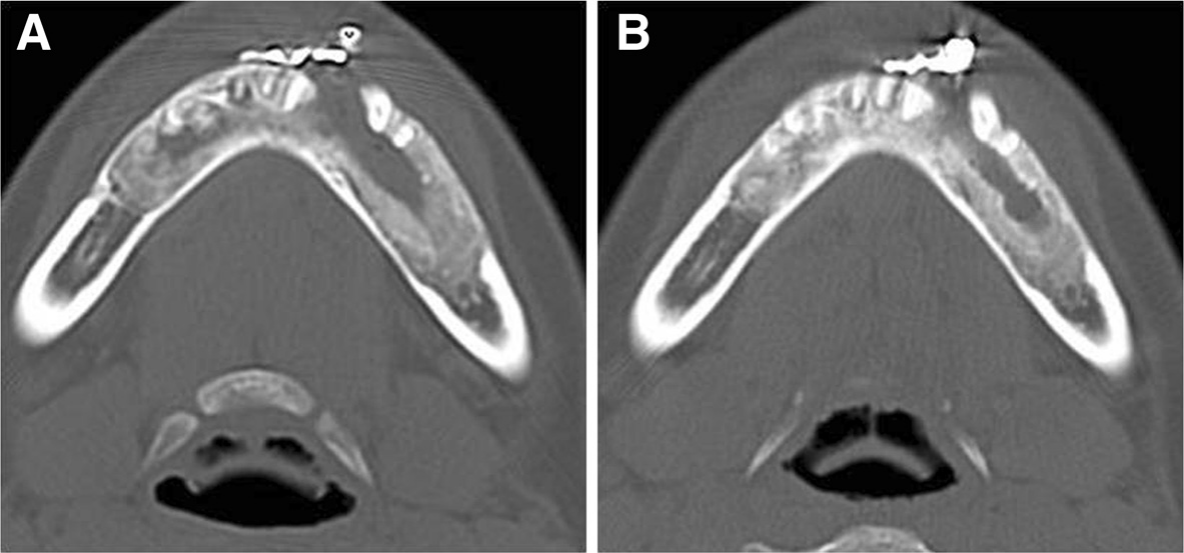
**a**, **b** Computerized tomogram images of mandible **a** 6 and **b** 12 months after surgery. Continuous bone formation is evident

After 1 month, one of the needles had fallen out of its hole, but the other was well maintained (Fig. 5). Continuous bone formation in the lesion was confirmed by CT scanning of the mandible every 6 months (Fig. 6a, b). Final enucleation and removal of the mini plates were performed 15 months after surgery.

**Fig. 7:**
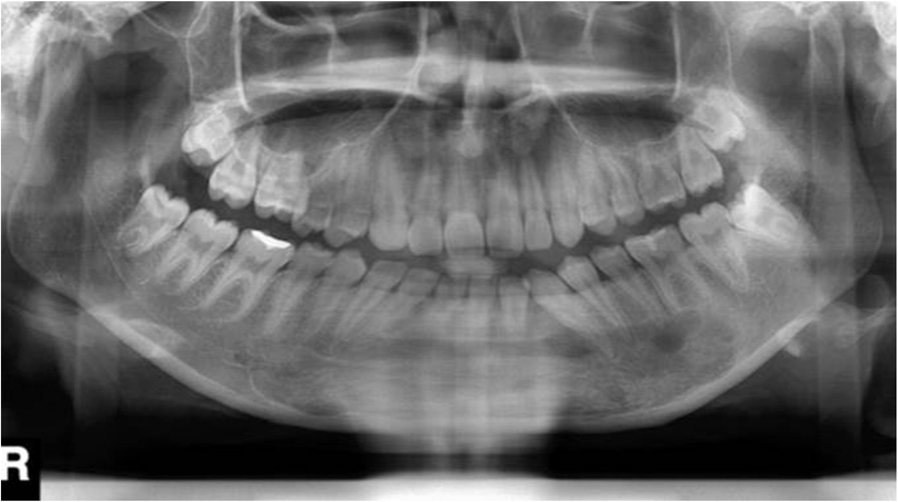
Panoramic radiographs after surgery. The dentition of the displaced teeth at the initial visit is restored somewhat to its original state

The dentition of the displaced teeth was restored somewhat to its original state by the completion of treatment (Fig. 7). Subsequently, following enucleation, continuous orthodontic treatment in our clinic was initiated.

### Discussion

For cystic pressure reduction and preservation of teeth, Thomas [4], in 1947, recommended decompression, which proceeds by formation of a small hole in the cystic cavity and insertion of a drain. The cystic cavity is gradually closed, both by relieving the pressure within it and by regular washing through the drain. Due to the fact that decompression/drainage is a long-term treatment, it is necessary to establish an environment for continuous drain maintenance and easy oral hygiene control. When using a rubber drain, suture maintenance is difficult, and periodic replacement is required; in any case, leaving in the same sutures over long durations increases the risk of infection.

In the present study, continuous self-cleaning with a 21G needle was performed by means of a decompressor attached to the mandible. The device was found to have been well attached, except for the fact that a drain had fallen out. Over the course of the follow-up period, no infection or cell necrosis around the fistula was observed. The mini plate must be attached to healthy bones, whose sites are determined prior to surgery by radiographic analysis (e.g., CT). Even if it has to be located above the lesion, it is still desirable to find as solid an area as possible.

During our follow-up period, we continuously performed CT radiography, observing gradual bone formation and cyst reduction. Moreover, the displaced teeth were recovered to the previous status, and no damage to the adjacent anatomical structures was evident. On this basis, it is considered that extensive cysts can be successfully treated by decompression if an appropriate decompressor is designed according to the lesion location and size and is well maintained over a long period of time.

**Fig. 8:**
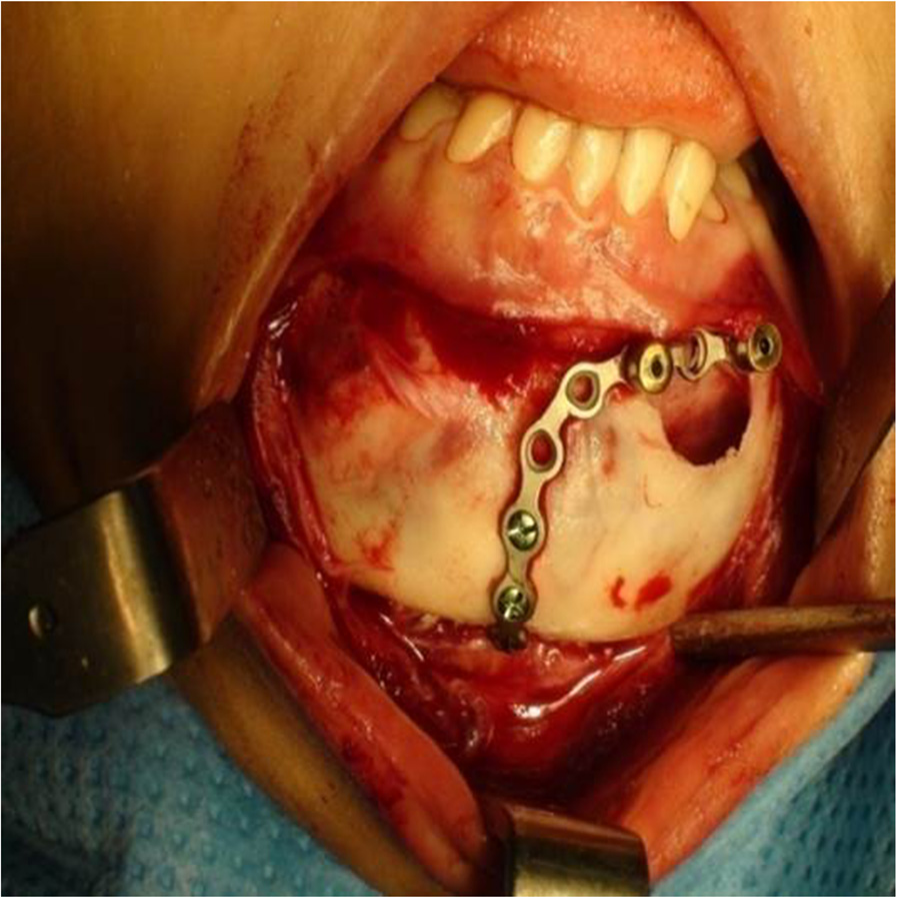
Attachment of first mini plate

However, repeated deformation of the mini plate can cause fracture, and attachment to the 16G spinal needle can fail due to resin breakage. In the present case, whereas the preoperative plan was to attach one mini plate with two holes to the lesion (Fig. 8), two mini plates were attached, because fracture occurred in the process of fitting the first. Where sufficient sound bone is lacking, applying two mini plates can be much more difficult than one, and proceeding with two can incur decompressor instability. Also, although a slightly more accurate decompressor can be produced by CT and surgical replica model, the cost burden on patients thereby is increased. In the present case, it was considered that one drain had fallen out after surgery, due to the contact area with the 16G spinal needle having become weak in the process of mini-plate deformation during surgery. Fracture in the contact area can be effectively prevented, therefore, only if the 16G spinal needle is attached after full bending of the mini plate.

Furthermore, this device, as it is custom-designed for the individual patient, should be prepared prior to surgery. Certainly, adequate presurgical preparations are necessary, particularly given the difficulty of finding an alternative in the event of device failure.

## Conclusions

When performing decompression, decompressor maintenance and infection prevention are essential. Although the decompressor can be maintained by fixation to the oral mucosa (with rubber tubes) or attachment to the teeth (with bands), customized decompressor design according to lesion location, size, and surrounding important anatomical structures is greatly preferred. In the present study, satisfactory decompression results, including reduction of mouth irritation, control of oral hygiene, reduction of lesion size, and long-term maintenance of the device, were achieved with a decompressor using the 16G spinal needle and mini plates without rubber tubes.

## Consent

Written informed consent was obtained from the parents of the patients for publication of this case presentation and any accompanying images. A copy of the written consent is available for review by the Editor-in-Chief of this journal.

## Abbreviations

G: gauge
CT: computerized tomogram
